# Phosphodiesterase 4 inhibitor combined with secukinumab relieves clinical symptoms in patients with psoriasis via regulating p38MAPK activation and the immune response inflammation

**DOI:** 10.4314/ahs.v25i1.35

**Published:** 2025-03

**Authors:** Qian Zhang, Zihan Zhou, Li Zhang, Xingxing Wang, Huani Zhao, Changzheng Wei, Min Zhao

**Affiliations:** 1 Department of Rheumatology and Immunology, Xi'an Fifth Hospital, Xi'an, China; 2 Department of Dermatology, Xi'an Fifth Hospital, Xi'an, China

**Keywords:** psoriasis, phosphodiesterase 4 inhibitor, secukinumab secukinumab, p38 MAPK

## Abstract

**Background:**

The purpose of the present study was to explore the effect of phosphodiesterase 4 (PDE-4) inhibitor combined with secukinumab monoclonal antibody on the activation level of p38 mitogen-activated protein kinases (p38MAPK) in psoriatic leukocytes.

**Methodology:**

The clinical data of 60 patients with psoriasis were retrospectively analyzed, and they were divided into a control group (secukinumab monotherapy, 30 cases) and a study group (PDE-4 inhibitory therapy). Agent combined with secukinumab treatment(30 cases), all were treated for 3 months. The Psoriasis Area and Severity Index (PASI), clinical symptom score, serum inflammatory factors, p38MAPK gene expression in lesion tissue, and clinical outcome were compared between the two groups.

**Results:**

After treatment, the PSAI score (t=5.051) and symptom score (t=14.102) of the study group were lower than those of the control group, and the relative expression of interleukin-6 (IL-6) (t=7.514) and p38MAPK (t=4.219), the relative expression of interleukin-17 (IL-17) (t=2.579) was lower than that of the control group. The total effective rate in the study group (83.33% vs. 60.00%) was higher than that in the control group.

**Conclusion:**

Secukinumab along with phosphodiesterase 4 inhibitors reduces p38MAPK activation, and improves immune response, inflammation, and clinical symptoms in patients with psoriasis.

## Introduction

Psoriasis is a refractory and extremely strong chronic inflammatory disease of the skin. A large number of studies have confirmed that chronic inflammatory immune responses to the skin are the main factors affecting the progression of psoriasis, but the pathogenesis of the disease has not been fully clarified^1^,^2^. It is believed that the biochemical reaction mediated by CD4 + T cells Th17 plays a crucial role in the development and occurrence of psoriasis and that this biochemical reaction activates p38 MAPK signaling to increase immune factors expression in patients and promote the differentiation of T cells into Th17, which in turn accelerates the patient's disease deterioration^3^.

For a long time, topical drugs, sequential therapy, phototherapy, retinoids, and immunomodulatory oral drugs have been the main ways of clinical treatment of psoriasis, but due to the extremely repetitive nature of psoriasis, long-term medication does not respond much, and the increase in drug resistance will also affect the treatment outcome of patients^4^. With the development of biotechnology, in recent years, the use of small molecule targeted drugs and biological agents has provided a new drug option for the treatment of psoriasis, and the effect of phosphodiesterase 4 (PDE-4) inhibitors and secukinumab secukinumab in the treatment of psoriasis has received the recognition of clinical scholars^5^,^6^,^7^. The pathogenesis of psoriasis involves an aberrant immune response, which leads to the activation of various signaling pathways in the skin cells, resulting in the development of characteristic symptoms such as erythematous plaques with silver scales, itching, and pain. One such signaling pathway that has been implicated in the pathogenesis of psoriasis is the p38MAPK pathway. p38MAPK (mitogen-activated protein kinase) is a serine/threonine kinase that is activated in response to various stress stimuli, including pro-inflammatory cytokines, oxidative stress, and UV radiation. Activation of p38MAPK has been shown to regulate the production of pro-inflammatory cytokines such as TNF-α and IL-17, which are key mediators of inflammation in psoriasis.

Studies have reported elevated levels of p38MAPK activation in the skin of psoriasis patients, suggesting that this pathway may play a critical role in the development of psoriatic lesions. Inhibition of p38MAPK has been shown to reduce inflammation and improve psoriatic skin lesions in preclinical models, indicating its potential as a therapeutic target. There has been no detailed report on the effect of phosphodiesterase 4 inhibitors on p38 MAPK in psoriasis patients in clinical practice, given that this study investigated the effect of phosphodiesterase 4 inhibitors combined with secukinumab secucizumab on p38 MAPK activation levels in psoriatic leukocytes.

## Patients and Methods

### Patients

The clinical data of 60 patients with psoriasis in our hospital from January 2020 to December 2021 were analyzed. The patients were divided into a control group (treated with scutellarin, 30 cases) and study group (treated with PDE-4 inhibitor combined with scutellarin, 30 cases) according to the differences in medication regimens. Inclusion criteria 1. Patients met the diagnostic criteria of psoriasis in the Chinese Guidelines for the Diagnosis and Treatment of Psoriasis (2018 Complete Edition)78; 2. Patients received phosphodiesterase 4 inhibitors (crisaborolecloborol) or secukinumab scotuzumab treatment in our hospital; 3. Patients received a p38 MAPK examination and blood biochemical indicators examination and the report was complete. Exclusion criteria: 1. Patients combined with other skin diseases; 2. Patients combined with other immunodeficiency diseases; 3. Patients with primary organ function injury; 4. Patients with coagulation dysfunction; 5. Patients with traumatic craniocerebral/limb injury; 6. Patients had received other treatment; 7. There were serious mental/psychological diseases. This study was approved by the ethical committee of Xi'an Fifth Hospital(NO.XAF202001001). All patients signed the relevant consent forms before the study was conducted.

### Methods

All patients with psoriasis should pay attention to diet control and ensure that the skin of the affected area was clean and dry during treatment. The control group was given secukinumab Sikuzumab Iinjection (1ml: 150 mg, production batch number 20191008, registration certificate number S20190023, Novartis Pharmaceuticals), 300 mg/(times · week) subcutaneous injection from the first week to the fourth week of diagnosis, and then changed to 300 mg/(times · 4 weeks) for 3 months; at the same time, the affected area was externally applied with calcipotriol ointment (0.75 mg: 15 g, production batch number 20190513, approval number H20160070, Leo Pharmaceuticals).

An appropriate amount was applied to the clean affected area twice a day, with a total weekly dosage of ≤ 100 g for 3 months. The study group received treatment with secukinumab scutuzumab combined with clarithromycin (PDE-4 inhibitor), calcipotriol ointment was discontinued and clarithromycin ointment (30 g, production batch number 20191116, GYZZ HJ20200022, Famasiapuqiang Pharmaceutical) was given, and an appropriate amount was applied to the clean affected area twice a day for 3 months.

#### Observation indicators

(1) **Symptom score:** Before and after treatment, the psoriasis area and severity index (PASI) score was used to evaluate the effect of symptom improvement^8^ improvement^9^. The total score of this scale was 72 points, indicating that the more severe the symptoms; meanwhile, the severity of symptoms such as skin lesion, skin lesion color, dry skin lesion, desquamation, or with itching (asymptomatic ∼ severe in turn scored 0 ∼ 6 points) was scored by referring to the Chinese Guidelines for the Diagnosis and Treatment of Psoriasis (2018 Complete Edition)78.

The total symptom score was the weighted average of each symptom score, with a total score of 6 points. (2) Clinical efficacy: The efficacy was evaluated after 3 months of treatment, and the efficacy criteria: (1) Significantly effective: PASI ≤ 30 points and the patient's skin lesion symptoms were significantly improved, and the skin status of the skin lesion site was good; (2) Effective: 30 points < PASI ≤ 40 points, the skin lesion was improved and the skin lesion of the affected area basically returned to normal; (3) Ineffective: did not meet the effective or significantly effective criteria; Total effective = significantly effective + effective. (3) Laboratory parameters: Serum interleukin-6 and 17 (IL-6/17, IL-6/17) were measured by ELISA assay enzyme immunoassay before and after treatment; skin tissues were collected from the affected area of patients and p38 MAPK gene expression was detected by polymerase chain reaction (PCR) (p38 MAPK primer sequence 5′-3′: R GTCCTGTTCTGTCGCGATF, F CTCCATAATGGCCGAGCT), and the relative gene expression was calculated to analyze the p38 MAPK activation level in patients^9^ patients^10^. (4) Adverse reactions: Skin tingling, erythema, itching, and other adverse reactions were compared between the two groups.

### Statistical analysis

Statistical Product and Service Solutions (SPSS) 23.0 (IBM, Armonk, NY, USA) was applied for statistical analysis. Independent sample t-test was used for comparison between groups for measurement data obeying normal distribution, and independent sample t-test was used for comparison within groups, all expressed as (x±s). Count data were tested by *χ*2 and expressed as a rate (%), P<0.05 indicates a statistical difference.

## Results

### Baseline data between the two groups

There was no significant difference in age (t = 0.423), disease duration (t = 0.362), body mass index (t = 0.175), gender (X2 = 0.071), and lesion site (X2 = 0.125) between the two groups (P > 0.05) (Table 1).

**Table 1:** Comparison of baseline data between the two groups

Group	N	Age (years)	Disease course (months)	Body mass index (kg/m^2^)	Gender [n, (%)]		Lesion Site [n, (%)]		

					Male	Female	Extremities	Extremities + head	Other
Study group	30	42.46 ± 3.82	19.13 ± 2.16	24.46 ± 4.17	19 (63.33)	11 (36.67)	15 (50.00)	10 (33.33)	5 (16.67)
Control group	30	42.53 ± 3.67	18.95 ± 2.07	24.65 ± 4.04	18 (60.00)	12 (40.00)	14 (46.67)	10 (33.33)	6 (20.00)
t/x^2^		0.423	0.362	0.175	0.071		0.125		
P		0.659	0.716	0.862	0.791		0.939		

Clinical symptoms between the two groups. After treatment, the PSAI score (t = 5.051) and symptom score (t = 14.102) in the study group were lower than those in the control group (P < 0.05) (Table 2).

**Table 2:** Comparison of symptom scores between the two groups (x̅ ± s)

Group	N	PASI (points)		Symptom score (points)	

		Before treatment	Post Treatment	Before treatment	Post Treatment
Study group	30	58.13 ± 7.21	32.16 ± 4.15	4.11 ± 0.22	2.41 ± 0.19
Control group	30	58.05 ± 7.19	39.04 ± 6.20	4.08 ± 0.25	3.26 ± 0.27
t		0.043	5.051	0.493	14.102
P		0.966	< 0.001	0.624	< 0.001

Note: PASI, Psoriasis Area Severity Index

Clinical efficacy between the two groups. After treatment, the overall response rate in the study group (83.33% vs. 60.00%) was higher than that in the control group (X2 = 4.022, P < 0.05) (Table 3).

**Table 3:** Comparison of clinical efficacy between the two groups [n, (%)]

Group	N	Markedly effective	Effective	Invalid	Total Effective
Study group	30	15	10	5	25 (83.33)
Control group	30	10	8	12	18 (60.00)
X^2^					4.022
P					0.045

Laboratory parameters between the two groups. After treatment, the relative expressionlevel of IL-6 (t = 7.514), p38 MAPK (t = 4.219), and IL-17 (t = 2.579) and the relative expression of p38 MAPK in the study group were lower than those in the control group (P < 0.05) (Table 4).

**Table 4:** Comparison of IL-6, p38 MAPK relative expression and IL-17 levels between the two groups (x̅ ± s)

Group	IL-6 (pg/ml)		p38 MAPK (Relative expression)		IL-17 (pg/ml)	

	Before treatment	Post Treatment	Before treatment	Post Treatment	Before treatment	Post Treatment
Study group	144.65 ± 17.33	61.98 ± 7.66	0.77 ± 0.12	0.40 ± 0.08	78.15 ± 9.26	31.08 ± 5.75
Control group	146.71 ± 15.52	78.76 ± 8.13	0.75 ± 0.09	0.55 ± 0.07	77.87 ± 8.84	38.56 ± 6.26
t	1.351	7.514	0.721	4.219	0.174	2.579
P	0.182	< 0.001	0.474	< 0.001	0.863	0.013

Note: IL-6, Interleukin-6; IL-17, Interleukin-17

Adverse reactions between the two groups. There was no significant difference in the adverse reaction rate between the two groups (16.67% vs. 10.00%) (x2 = 0.144, P > 0.05) (Table 5).

**Table 5:** Comparison of adverse reactions between the two groups [n, (%)]

Group	N	Skin tingling	Pruritus	Erythema	Total adverse reactions
Study group	30	1	2	1	5 (16.67)
Control group	30	1	1	1	3 (10.00)
X^2^					0.144
P					0.704

## Discussion

Psoriasis is a refractory and extremely strong chronic inflammatory disease of the skin. Patients mainly present with skin erythema with white scales, mostly in the limbs and scalp, and other easily exposed parts. Some patients may suffer from pruritus, which can seriously affect the aesthetics of the skin and the quality of life of patients^10^ patients^11^. Pruritus has been found to be associated with decreased quality of life in multiple studies^10^ studies^11^,^11^,^12^. It can have a negative impact on physical, emotional, and social well-being, leading to depression, anxiety, social isolation, and decreased productivity. Patients with chronic pruritus may also experience financial burdens due to the cost of treatments and lost workdays. Management of pruritus is essential to improve the quality of life of patients. Treatment options include topical and systemic medications, phototherapy, and counseling. Addressing the underlying cause of pruritus is also crucial in managing the symptoms and improving quality of life. Psoriasis has a long course of the disease and is extremely repetitive. Patients also have a high risk of recurrence after systemic treatment. Early patients have no obvious symptoms. If not treated in time, it may lead to multiple systemic involvement and arthritis, general malaise, and other complications, which is extremely harmful. The pathogenesis of psoriasis is complex, and chronic inflammatory immune responses in the skin are considered to be one of the main pathogenic causes of this disease^12^ disease^13^,^13^,^14^. Studies have shown that CD4 + T cells Th17 can affect the expression of serum inflammatory factors in patients with psoriasis, of which IL-6 and IL-17 play an important role in the occurrence and development of psoriasis^14^ psoriasis^15^. Th17 cell-mediated biochemical reaction can promote the secretion of IL-17 and activate the p38 MAPK signaling pathway to up-regulate the expression of IL-6, while IL-6 can stimulate primitive T cells to differentiate into Th17 cells to form circulatory effects and accelerate the occurrence and development of psoriasis. secukinumab Stacutuzumab and crisaborolecloborol can inhibit the expression of PDE-4 and IL-17 in the human body, respectively, and then attenuate Th17 cell-mediated immune response so as to relieve the clinical symptoms of psoriasis patients and achieve control and therapeutic effects.

In this study, we investigated and analyzed the clinical data of 60 patients with psoriasis, and the results showed that the symptoms of all patients were improved after treatment with secukinumab scutellizumab or cliborol, and the relative expression of IL-6, p38 MAPK, and IL-17 expression was significantly down-regulated. Further analysis was performed on the treatment results of the two groups. The results showed that the clinical efficacy of the study group receiving the combination of secukinumab scutellizumab and crisaborolecloborol was better than that of the control group, and the relative expression levels of IL-6, p38 MAPK, and IL-17 after treatment were lower than those of the control group, indicating that the combination of secukinumab scutellizumab and crisaborolecloborol could further improve the expression levels of IL-6, p38 MAPK, and IL-17 in patients with psoriasis and improve the clinical treatment effect of patients. The reason for analysis, on the one hand, is due to the specific neutralizing effect of secukinumab secukinumab on human IL-17A, which can neutralize the biological activity of IL-17A and then regulate the expression of IL-17 in psoriasis patients, inhibit the abnormal activation of p38 MAPK signaling pathway by IL-17, indirectly down-regulate the expression of IL-6, and improve the immune inflammatory response in psoriasis patients15patients16. On the other hand, PDE is due to its ability to hydrolyze intracellular cyclic adenosine monophosphate (cAMP) in the lesional skin of psoriasis patients, which in turn leads to the inability of normal activation of the cAMP-PKA pathway, attenuates the inhibitory effect on the synthesis of inflammatory factors such as nuclear factor-kB and nuclear factor of activated T cells (NFAT), and aggravates the condition of psoriasis patients; while PDE-4 inhibitors can highly selectively inhibit PDE-4 activity in psoriasis patients, block the hydrolysis of cAMP by PDE-4, weaken the immune-inflammatory response of Th17 cells, and then reduce IL-17 secreted by mature Th17 cells and reduce p38 MAPK activation levels and IL-6 expression levels^16^ levels^17^. This article still has several limitations. For example, this study was conducted on patients with the only disease of psoriasis, and does the age of the patient has an effect on the efficacy of the treatment. Besides, the sample size included in this study was small and this study was a single-center study.

## Conclusion

Phosphodiesterase 4 inhibitors combined with secukinumab scotuzumab have good efficacy in the treatment of psoriasis, can reduce the level of p38 MAPK activation in patients with psoriasis, improve immune response to inflammation and clinical symptoms, and has the value of the clinical application.

## Data Availability

The datasets used and analyzed during the current study are available from the corresponding author upon reasonable request.
